# Supplementary material for: Meta-Analysis and Gene Set Enrichment Relative to ER Status Reveal Elevated Activity of MYC and E2F in the “Basal” Breast Cancer Subgroup
Source: PLoS One. 2009 Mar 9;4(3):e4710. doi: 10.1371/journal.pone.0004710 (PMC2650420; doi:10.1371/journal.pone.0004710)
Supplement: Dataset S1 — GSEA results from first screen. The file contains a zipped archive of the HTML results of the second GSEA screen. Reports for enriched or depleted datasets may be accessed from the “index.html” link within the my_analysis.GseaPreranked.1219975950625.rpt.zip ZIP archive. Enrichment plots are available for each of the 50 most enriched and depleted gene sets. (13.32 MB ZIP) [file pone.0004710.s001.zip › my_analysis.GseaPreranked.1219975950625.rpt.zip!//CHR5Q13.html]

Details for gene set CHR5Q13[GSEA]

|  || Dataset | zSco.ERneg.to.ERpos\_collapsed |
| Phenotype | NoPhenotypeAvailable |
| Upregulated in class | na\_neg |
| GeneSet | CHR5Q13 |
| Enrichment Score (ES) | -0.62278116 |
| Normalized Enrichment Score (NES) | -2.245652 |
| Nominal p-value | 0.0 |
| FDR q-value | 4.7390308E-4 |
| FWER p-Value | 0.0070 |
Table: GSEA Results Summary

  

Fig 1: Enrichment plot: CHR5Q13      
 Profile of the Running ES Score & Positions of GeneSet Members on the Rank Ordered List

  

| PROBE | GENE SYMBOL | GENE\_TITLE | RANK IN GENE LIST | RANK METRIC SCORE | RUNNING ES | CORE ENRICHMENT || 1 | SV2C |  |  | 1884 | 2.619 | -0.1060 | No |
| 2 | AGGF1 |  |  | 2336 | 2.299 | -0.1086 | No |
| 3 | CKMT2 |  |  | 2353 | 2.284 | -0.0787 | No |
| 4 | MAP1B |  |  | 2569 | 2.162 | -0.0654 | No |
| 5 | PIK3R1 |  |  | 3330 | 1.780 | -0.0983 | No |
| 6 | BHMT |  |  | 5258 | 1.062 | -0.2288 | No |
| 7 | BHMT2 |  |  | 5544 | 0.960 | -0.2371 | No |
| 8 | ADAMTS6 |  |  | 6409 | 0.681 | -0.2929 | No |
| 9 | LHFPL2 |  |  | 7616 | 0.263 | -0.3800 | No |
| 10 | F2RL1 |  |  | 8320 | -0.005 | -0.4328 | No |
| 11 | TNPO1 |  |  | 8912 | -0.267 | -0.4737 | No |
| 12 | GPR98 |  |  | 8941 | -0.282 | -0.4719 | No |
| 13 | ENC1 |  |  | 9363 | -0.491 | -0.4969 | No |
| 14 | KIAA0888 |  |  | 9760 | -0.701 | -0.5171 | No |
| 15 | PTCD2 |  |  | 9837 | -0.747 | -0.5127 | No |
| 16 | CARTPT |  |  | 10510 | -1.172 | -0.5472 | No |
| 17 | HMGCR |  |  | 11515 | -2.051 | -0.5948 | Yes |
| 18 | SCAMP1 |  |  | 11847 | -2.367 | -0.5874 | Yes |
| 19 | BTF3 |  |  | 12059 | -2.650 | -0.5672 | Yes |
| 20 | F2RL2 |  |  | 12474 | -3.220 | -0.5544 | Yes |
| 21 | DKFZP686M0199 |  |  | 12597 | -3.428 | -0.5168 | Yes |
| 22 | COL4A3BP |  |  | 12900 | -4.151 | -0.4830 | Yes |
| 23 | TINP1 |  |  | 12956 | -4.342 | -0.4279 | Yes |
| 24 | MRPS27 |  |  | 12964 | -4.371 | -0.3688 | Yes |
| 25 | HEXB |  |  | 12967 | -4.383 | -0.3092 | Yes |
| 26 | CCNH |  |  | 13015 | -4.513 | -0.2512 | Yes |
| 27 | RASA1 |  |  | 13063 | -4.690 | -0.1908 | Yes |
| 28 | RAD17 |  |  | 13147 | -5.016 | -0.1286 | Yes |
| 29 | F2R |  |  | 13158 | -5.095 | -0.0599 | Yes |
| 30 | SMA3 |  |  | 13185 | -5.279 | 0.0102 | Yes |
Table: GSEA details [plain text format]

  

Fig 2: CHR5Q13: Random ES distribution      
 Gene set null distribution of ES for **CHR5Q13**
